# Understanding vigilance and its decrement: theoretical, contextual, and neural insights

**DOI:** 10.3389/fcogn.2025.1617561

**Published:** 2025-09-22

**Authors:** Klara Hemmerich, Fernando G. Luna, Elisa Martín-Arévalo, Juan Lupiáñez

**Affiliations:** ^1^Department of Psychology and Cognitive Science, University of Trento, Rovereto, Italy; ^2^Department of Experimental Psychology and Mind, Brain, and Behavior Research Center (CIMCYC), Universidad de Granada, Granada, Spain; ^3^Facultad de Psicología, Universidad Nacional de Córdoba, Córdoba, Argentina

**Keywords:** vigilance, vigilance decrement, sustained attention, arousal, resources

## Abstract

The vigilance decrement refers to the gradual decline in the ability to monitor the environment and detect rare but critical stimuli over time. This phenomenon occurs in many everyday situations and work environments and may be exacerbated by brain damage or developmental disorders. However, despite its seeming omnipresence, the exact meaning of “vigilance” and vigilance decrement is often unclear, with the term “vigilance” frequently used interchangeably with related concepts such as arousal, alertness, or sustained attention. This narrative review seeks to clarify this conceptual overlap, offering a precise definition of vigilance, whilst separating it from these other phenomena. Furthermore, this narrative review also provides a detailed account of some of the factors that modulate vigilance performance, as well as an overview of current theories that explain its frequent and progressive decrement over time. Lastly, it highlights the most relevant structural and electrophysiological correlates of its proper functioning. By integrating these insights, a more refined understanding of vigilance and its decrement may emerge, helping to unify future research findings and facilitate the development of interventions to mitigate its effects.

## 1 Introduction: historic and current definitions of vigilance

Keeping attention focused is essential for human cognition, and thus, also for interacting with the external world. Vigilance is exerted when the focus of attention is to be maintained for extended periods, eliciting a low level of responses. However, despite the importance of maintaining adequate performance (i.e., detecting and responding to these rare stimuli), vigilance frequently and unwillingly declines over time—a phenomenon that is well-documented in scientific literature and a common occurrence in everyday life. For instance, during a lecture we may notice that our ability to engage with new information diminishes over time. Later on, while driving home, we may miss exits or turns, overlook a pedestrian about to cross the street, or fail to notice that a traffic light has turned red in time. While the consequences of the vigilance decrement might go mostly unnoticed in the first scenario, they can be dire in the second one. In fact, inattention causes almost a third of fatal road accidents (Wundersitz, 2019). Human errors related to attentional failures are reported in other realms as well, including railway (Edkins and Pollock, 1997) and aviation accidents (Kharoufah et al., 2018), missed threats at security screenings (Krüger and Suchan, 2015; Meuter and Lacherez, 2016; Näsholm et al., 2014), or medical errors (Barger et al., 2006; Caruso, 2014). Moreover, developmental or lesion-induced alterations in brain functioning can impair the ability to maintain vigilance, hindering a correct interaction with the environment and the proper functioning of higher-order cognitive processes (Fish et al., 2017; Zimmermann and Leclercq, 2002). Given its implication in daily life and clinical settings, it is crucial to further investigate the vigilance decrement to better understand its causes, modulating factors, and potential countermeasures. To this end, the present narrative review aims to provide an overview of the historical development of the concept, propose a more unified definition of vigilance, and examine the most commonly used explanatory theories and proposed neural correlates.

### 1.1 Brief history of vigilance and its decrement

The term vigilance stems from the Latin *vigil* or *vigilare*, referring to being awake, watchful, or alert. The diverse meanings attributed to the concept's root may actually foreshadow the wide range of attributes it still holds today. The first conception of relevance stems from the medical field, where it was not considered a cognitive skill nor attributed to consciousness (Klösch et al., 2022), but rather to the organism's ability to reorganize itself in the process of restoration from damage or trauma (Head, 1923). Head's conceptualization, formulated a century ago, viewed vigilance as a sign of responsiveness from the organism in its recuperation process (e.g., reflex upon stimulation). Despite this more medically oriented framing, his assertion that “*when vigilance is high, the body is more prepared to respond to an effective stimulus with a more or less appropriate reaction*” (Head, 1923), has carried over into later conceptualizations of arousal, which plays an important role in vigilance.

Twenty years later, Norman Mackworth refined the concept of vigilance in terms more relevant for cognition as a “*psychological readiness to perceive and respond, a process which, unlike attention, need not necessarily be consciously experienced*” (Mackworth, 1948). Mackworth was commissioned in 1943 to study why operators from the British Air Force missed crucial detections of German submarines in their airborne radars. He examined the working conditions of these operators and then replicated the environment's characteristics in a laboratory setting to systematically encompass the phenomenon at hand. For this purpose, the Mackworth Clock Task (MCT) was designed, imitating the sweeping radial motion of the radars: a fine line akin to a clock hand was projected onto a white background in a monotone setting. Observers had to keep their attention on the clock hand to detect the occurrence of an infrequent signal: a double jump of the clock handle. Through this experiment, the vigilance decrement was characterized by its now distinctive curve: during a 2-h watch, the “operators” would face a steep drop in their detection accuracy in the first 30 min, followed by a more steady decline (Mackworth, 1948).

Since Mackworth's first experimental investigation of the vigilance decrement, the phenomenon has received heightened interest, mobilizing extensive efforts to further its understanding. However, the current literature still lacks a firm grasp on the factors most relevant to determining the magnitude and time-course of the vigilance decrement, a unified theory accounting for the diverse manifestations of the vigilance decrement in different contexts, a clear unitary definition within attention taxonomy, or unambiguous neural correlates. Nevertheless, in the following sections, we will delve into what we know about these aspects up to now.

### 1.2 Developing an unambiguous definition of vigilance

#### 1.2.1 Vigilance as an independent construct

A challenge imposed by the concept of vigilance is its varied meanings and applications across different fields. In neurophysiology or psychiatry, the meaning of vigilance is more tied in with physiological, either healthy or pathological, fluctuations of arousal. Neurophysiologists place vigilance as an intermediate state within the sleep-wake cycle, which can range from hypervigilance (over-excited), to vigilant (relaxed awake state), to a drowsy or hypo-vigilant, and a sub-vigilant state that transitions into sleep (Klösch et al., 2022; Oken et al., 2006). Psychiatrists, instead, refer to abnormal states of vigilance. On the one hand, they consider hypervigilance as a heightened attentiveness and response toward the environment, that may lead to perceiving innocuous stimuli as threats and is often observed as a clinical symptom of post-traumatic stress disorder (American Psychiatric Association, 2013; Oken et al., 2006). On the other extreme, hypo-vigilance is considered as a dampened responsiveness toward the environment, observable in depression (Weinberg and Harper, 1993). This shows how diversely the term “vigilance” is defined and used across disciplines. However, even within cognitive psychology and neuroscience, the term lacks a clear, consistent definition and is often used interchangeably with related phenomena such as arousal, alertness, and sustained attention. This section aims to disentangle a less ambiguous definition of vigilance by more clearly separating it from these overlapping but relatively distinct phenomena.

**Figure 1:**
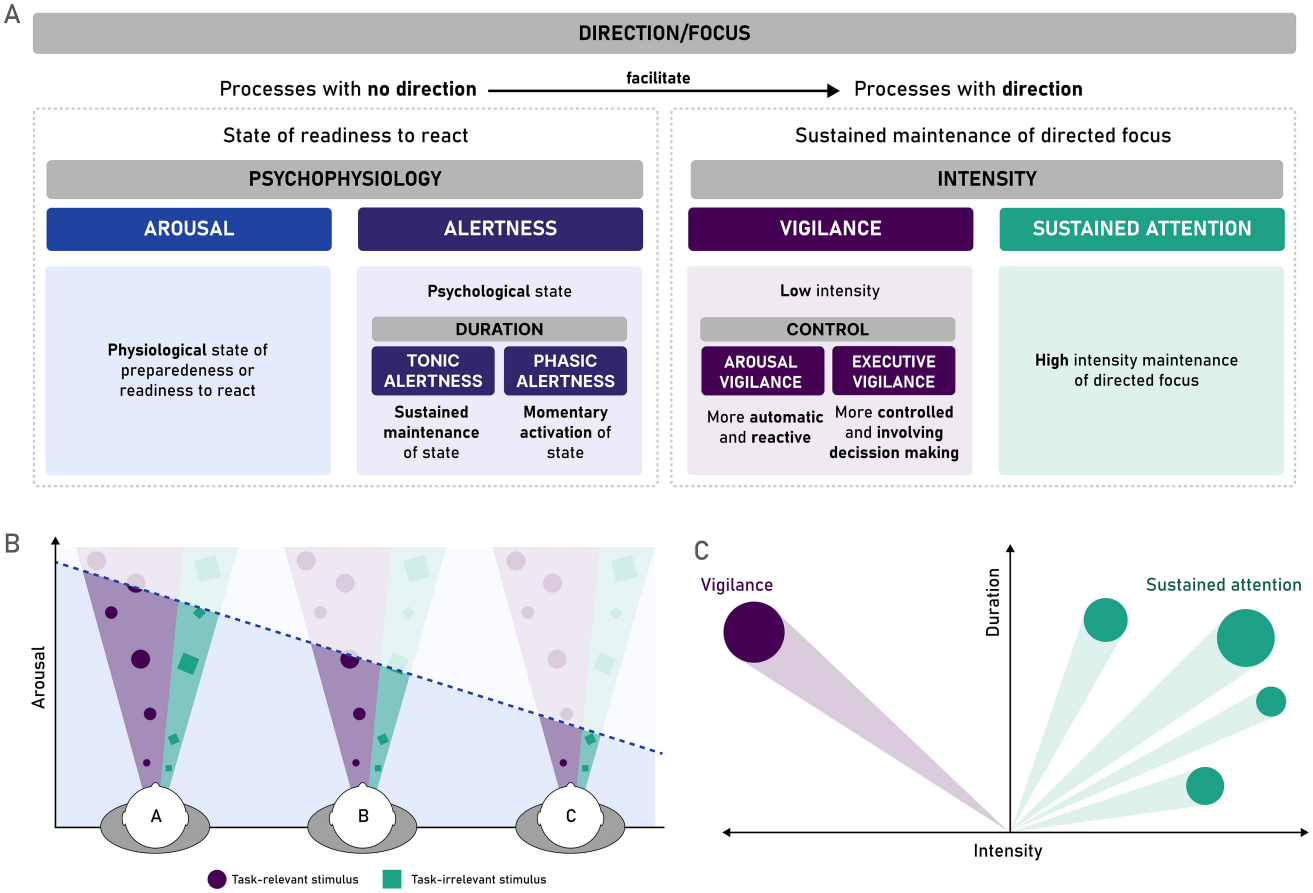
Arousal, alertness, vigilance, and sustained attention as interrelated but differentiable phenomena. **(A)** A proposed differentiation of different processes and phenomena that are often used interchangeably with vigilance. Non-directional processes, arousal (a physiological state of readiness to react) and alertness (the psychological counterpart of this state), facilitate other more complex processes, such as those requiring a specific direction of the attentional focus over an extended period, such as vigilance and sustained attention. These two processes can be distinguished based on the intensity required by this focus, distinguishing low intensity processes (vigilance) from high intensity ones (sustained attention). **(B)** Arousal, understood as a general level of cortical activation, can lead to suboptimal inputs of information when activation levels are too high (hyper-arousal, person A) or too low (hypo-arousal, person C). With intermediate levels, the input of information is ideal (person B). Adapted from Esterman and Rothlein (2019). **(C)** Vigilance and sustained attention share that they both have a specific focus or direction (stimulus or stimuli, or task), but are distinct in terms of the intensity of or amplitude of the focus needed to maintain good performance at detecting the specific stimulus or performing the specific task.

A first broad distinction can be made in terms of the attentional component of *direction* or *focus*, i.e., cortical activity that is directed or focused toward a specific stimulus, task, or purpose (van Schie et al., 2021). In this sense, direction refers not just to readiness to respond but to the continuous selection and monitoring of specific, task-relevant inputs over time, which goes beyond the more global readiness state that may facilitate fast responses or anticipate the selection of motor responses. This distinction based on direction, as depicted in Figure 1A, permits to jointly categorize arousal and alertness as processes attributed to cortical activity without a specific direction or selectivity, and to differentiate them from processes that do require a direction, such as vigilance and sustained attention.

Regarding processes *with no direction*, both arousal and alertness refer to a more generalized readiness to react, that subserves and facilitates other more complex cognitive processes. However, the two phenomena can be distinguished based on the level at which this readiness to react manifests. Arousal can be understood as a general physiological state of being awake or reactive to the environment, more in line with Head's original concept of vigilance (Head, 1923). It encompasses different levels of consciousness, from drowsiness or hypo-arousal states (such as sleep) to the opposite extreme of hyper-arousal (Aston-Jones and Cohen, 2005; Klösch et al., 2022; Unsworth and Robison, 2017). Arousal has been considered a pre-requisite for adequate cognitive processing (Aston-Jones and Cohen, 2005), and specifically for vigilance or sustained attention performance (Esterman and Rothlein, 2019). The monitoring required by vigilance tasks requires a certain level of cortical activation that depends on arousal (Sarter et al., 2001). In fact, it is known that the effects of arousal on these attentional processes are modulated by the effects of norepinephrine (NE) released by the locus coeruleus (LC): as low or high locus coeruleus activity is associated with poor task performance, either due to low task engagement (hypo-arousal) or over-active but nonspecific task engagement (hyper-arousal), respectively (Esterman and Rothlein, 2019). Thus, as depicted in Figure 1B, a state of preparedness or readiness to react at the physiological level—when properly balanced—can be considered as a filter that allows the input of adequate levels of task-relevant and task-irrelevant information.

Alertness, on the other hand, supported by arousal on the physiological level, refers to a psychological dimension of this state of readiness to react and respond to the environment—more in line with Mackworth's (1948) above-mentioned definition of vigilance. Alertness depends on an optimal level of arousal, allowing adequate sensitivity to incoming stimuli (Posner, 2008). Alertness has additionally been subdivided into a tonic component, referring to slow changes associated with circadian rhythms, where the level of cortical activity allowing responsiveness to the environment is sustained for longer periods and experiences slow fluctuations over time (Posner, 2008; Sturm and Willmes, 2001); and a phasic component, which alludes to quicker or momentaneous switches into this state of readiness, that occur in response to an external cue or stimulus or self-initiated due to the expectation of a stimulus (Petersen and Posner, 2012; Sturm and Willmes, 2001). Although alertness is understood as a general, non-specific state of readiness, its expression within task contexts can sometimes appear directional. For instance, when tasks include specific cues or known response mappings, they may produce preparatory neural activity, such as electrophysiological contingent negative variation (CVN; Walter et al., 1964), which reflects task-induced anticipation (Van Boxtel and Böcker, 2004; Walter et al., 1964), and thus heightened cortical activity for a specific sensory modality or motor response. However, this directionality should not be attributed to alertness itself, but rather to more voluntary mechanisms such as temporal orienting (Correa et al., 2006; Nobre et al., 2007), or task-induced constraints that shape how alertness is expressed at the behavioral and neurophysiological level (Sarter et al., 2001). Furthermore, classic models of attention state that alertness remains fundamentally non-selective and diffuse, facilitating more targeted attentional processes such as orienting or vigilance (Petersen and Posner, 2012; Posner, 2008; Sturm and Willmes, 2001).

Thus, while tonic alertness has sometimes been equated to vigilance (Posner, 2008) and sustained attention, they would differ in the lack of direction or selective focus associated with vigilance, still reflecting a more general and diffuse state of preparation. Yet, despite this fundamental non-selectivity of alertness, recent accounts have argued that in certain situations, such as under increased urgency or emotional saliency, high (phasic) alertness may temporarily bias or override the selection of actions or responses. For instance, Poth (2021) and Krause and Poth (2025) describe how external stimuli can sometimes override top-down control, triggering stimulus-driven actions that conflict with current intentions, or temporal expectancies (Cappucci et al., 2018). These phenomena suggest that under specific conditions, arousal and alertness may modulate the readiness of certain motor plans, leading to a context-dependent directionality in behavior. However, we propose that this directionality arises not from an inherent selectivity of arousal or alertness themselves, but rather from automatic interactions between these global readiness states and environmental affordances or task demands. In other words, arousal and alertness remain fundamentally non-specific states (Weinbach and Henik, 2012); although their behavioral expression can become more directional under certain contextual conditions, that override the current task goal with a more relevant one in the moment (e.g., survival).

The two processes *with direction*—vigilance and sustained attention—are often used interchangeably (Klösch et al., 2022; Oken et al., 2006; Sarter et al., 2001), as both require the focus of directed attention on a task over a prolonged period. However, one can distinguish between the two in terms of the *intensity* of information processing that is required (van Zomeren and Brouwer, 1994; Zimmermann and Leclercq, 2002): whereas vigilance would refer to the detection of infrequent (and potentially harder to detect) changes in the environment, sustained attention would require more active and ongoing processing toward a broader set of stimuli (Singh-Curry and Husain, 2009), as schematically depicted in Figure 1C. For example, vigilance, on the lower end of the intensity continuum, might involve driving down a long, straight highway with minimal traffic, where responding to external stimuli is rare (e.g., adjusting speed in accordance with a speed-limit change or braking when noticing that cars ahead are doing so). On the opposite end of the intensity continuum, sustained attention, exemplified by driving through city traffic at rush hour, requires constant attention to a rapidly changing and stimulating environment with potentially several different foci to be attended simultaneously (e.g., traffic lights, pedestrians about to cross the street, other cars, etc.). Importantly, intensity is not only dependent on the frequency of target stimuli, as it may also interact with the saliency of targets or the processing they require; where higher target saliency may facilitate detection (Helton and Warm, 2008; Smallwood, 2013). In cases of lower saliency and low task demands (e.g., simple detection), participants must sustain control settings for selective attention over long intervals with little immediate reward or reinforcement, a situation that may promote disengagement or exploratory shifts in attention unless sufficient task utility is maintained (Aston-Jones and Cohen, 2005).

Based on this differentiation from other phenomena, we can propose that vigilance may be defined as the ability to monitor the environment and detect rare but critical stimuli. This definition accurately reflects the directionality (cortical activity directed toward a specific critical stimulus or task) and the low intensity (rare appearance of the critical stimulus in the environment) of vigilance. It also aligns with the working definition used across several research projects authored or co-authored by the current authors (Cásedas et al., 2022; Hemmerich et al., 2023, 2024; Luna et al., 2021a, 2018, 2020, 2021c, 2022a,b; Román-Caballero et al., 2021; Sanchis et al., 2020). In line with this definition, the decrement of vigilance with time-on-task can be understood, not as a decline in arousal or alertness (although these processes subserve, and therefore, influence vigilance performance), but rather due to a loss of maintaining a continuous directional focus over a selected stimulus over time. Section 2 will cover theories that have been established to explain why the vigilance decrement takes place.

Finally, it is important to clarify the functional role we attribute to the concept of vigilance in our framework. While vigilance is often operationalized in terms of behavioral outcomes (e.g., target detection rates, reaction time variability, or performance decrements over time), our definition aims to go beyond the behavior itself, presenting vigilance as an explanatory construct, i.e., a latent cognitive state characterized by sustained and directed monitoring for infrequent but critical stimuli. This state is inferred from empirical observations but not reducible to them. Making this distinction is important for interpreting results: behavioral indicators are necessary to measure vigilance, but our goal is to understand the underlying process that gives rise to those observable patterns. The following section will briefly cover aspects related to the empirical observation of the construct, to highlight how the vigilance decrement manifests over time, and which tasks and specific measures have been used to identify it.

#### 1.2.2 Operationalizing vigilance and its decrement: tasks, measures, and time-course

Common paradigms used to assess vigilance have been summarized in Table 1, so as to give a better overview of how it is usually operationalized in research. Across the studies that employ these tasks, a certain overreliance on hit rates, accuracy measures, and simple reaction times (RTs) is noted. Some paradigms allow for the extraction of Signal Detection Theory (SDT) measures (Stanislaw and Todorov, 1999), that offer a more nuanced interpretation of performance. Specifically, the vigilance decrement may arise from a shift toward a more conservative response criterion (e.g., β) or a decline in perceptual sensitivity (e.g., d′). Recent research has highlighted the importance of disentangling these components. Thomson et al. (2016) argue that much of the observed vigilance decrement may be better accounted for by strategic shifts in response bias rather than by a true loss of perceptual sensitivity. In their view, motivational and effort-related factors, rather than purely sensory fatigue, drive participants to adopt a more conservative criterion to minimize errors. Luna et al. (2022b) further stress that the use of SDT measures can uncover hidden dynamics in attentional control: in some cases, sensitivity may retain stable while response bias fluctuates, revealing changes in cognitive control, motivation, or perceived task demands rather than perceptual degradation *per se*. Despite these insights, SDT metrics remain underutilized in many vigilance studies, limiting our ability to specify the cognitive processes underlying performance changes. Recent evidence inspecting psychometric curves have allowed identifying which SDT measures contribute most to the vigilance decrement. McCarley and Yamani (2021) observed that a shift toward a more conservative response criterion, decreased sensitivity, and increased attentional lapses were associated with the vigilance decrement. However, subsequent studies using the same approach have identified only the response bias and lapses as robust predictors of the vigilance decrement (Gyles et al., 2023; Román-Caballero et al., 2023).

**Table 1:** Tasks used to asses vigilance performance.

**Task**	**Description**	**Measures of vigilance decrement**
Mackworth Clock Test (MCT)	A clock hand jumps in regular steps, and participants detect occasional double jumps.	Miss rate (failure to detect double jumps), Hit rate, RT
Psychomotor Vigilance Task (PVT)	Participants respond as quickly as possible to a visual stimulus appearing at random intervals.	RT variability, number of lapses, number of false alarms
Sustained Attention to Response Task (SART)	Participants respond to frequent non-targets, whilst withholding responses for infrequent targets.	Commission errors (responding to target), omission errors (missing non-targets), RT and RT variability
Continuous Performance Task (CPT)	Participants respond to specific infrequent target letters or image sequences, ignoring non-targets.	Hits, false alarms, RT, d' (sensitivity), β (response criterion)
AX-CPT	Participants respond to an “X” only if preceded by an “A” cue.	Hit rate for AX trials, error rates in AY, BX, and BY trials, RTs by trial type, proactive control index
Gradual Onset CPT (gradCPT)	Participants continuously view images that gradually morph from one to another; responding to most of them, withholding responses for rare targets.	Commission errors, omission errors, RT variability, d'
Visual vigilance task	Participants monitor a visual stream for occasional critical signals (e.g., a slightly longer line).	Hits, misses, RT, false alarms
Auditory vigilance task	Akin to the prior task, but using tones (e.g., detect deviant tones in a stream).	Hits, misses, RT, false alarms
Oddball task	Participants respond to rare target stimuli among frequent standard stimuli.	RT and accuracy to targets, misses and false alarms

Most of the research that has attempted to establish a time-course of the vigilance decrement converges on the findings that rather than a steady linear decline with time-on-task, performance declines steeply in the early phases of a task and then plateaus or presents a less steep decline. This pattern was already evident in early experimental work, such as Mackworth's (1948) Clock Test, where declines in signal detection were most prominent within the first 30 min, followed by a steadier decline in the remainder of the full 2 h of the task. Teichner's (1974) review on vigilance studies also observes that performance in vigilance tasks generally declines early on in the task, highlighting the need to include fine-grained measures in the time-domain to adequately characterize the vigilance decrement. Further work has supported this general pattern: modeling approaches have frequently used an exponential function to characterize the time course of the performance decline (Helton et al., 2007; Parasuraman and Jiang, 2012; Warm et al., 2008). However, Parasuraman and Jiang (2012) also point out that, critically, the established patterns are often only observed when the data is analyzed at a group level, but that individual vigilance trajectories over time do rarely adjust well to such (e.g., exponential) fits, suggesting that this pattern may be a useful heuristic only at the group level. However, it does not necessarily capture the full heterogeneity in how vigilance fluctuates within and across individuals, as well as across tasks, task conditions, or assessed dependent variables. Surprisingly, the temporal aspect of vigilance is severely under-reported, with many studies presenting only mean aggregated values across the task, while not investigating how performance or SDT measures may fluctuate over time (e.g., at the block or trial-level), further obscuring an adequate understanding of a precise time-course of vigilance.

Lastly, more critical accounts of the vigilance decrement have suggested that it may, in fact, be an iatrogenic phenomenon (Hancock, 2013, 2017). Hancock argues that the decrement arises due to the artificial imposition of the vigil itself, claiming it is more a product of laboratory design than a phenomenon with real-world validity. This critique also raises the issue of distinguishing empirical observations of a phenomenon from their mechanistic explanation. In the case of vigilance, however, there is a certain overlap in the mechanistic or theoretical definition of the construct and its operationalization. In this sense, direction and intensity are both core components of the mechanisms defining vigilance, as well as aspects elicited by vigilance tasks. While critical points such as those raised by Hancock (2013) are valuable reminders to continuously interrogate the assumptions behind experimental paradigms, we argue that the core function of sustained environmental monitoring remains essential in many real-life settings. Indeed, current empirical evidence supports that vigilance-like performance failures do occur outside of controlled laboratory conditions. For example, studies on automated driving have consistently shown that drivers' ability to detect critical events declines over time, even in realistic, safety-critical contexts (Biondi et al., 2024; McWilliams and Ward, 2021). Similar decrements have been observed in surveillance and military monitoring tasks (Wohleber et al., 2019), supporting the view that the vigilance decrement reflects an actual impediment to directing the attentional focus on critical targets in real-life scenarios. That said, Hancock's (2013) critique also raises an important point about the ecological validity of experimental findings. Enhancing ecological validity would not only clarify the generalizability of lab-based results but also support the development of protocols better suited to real-world and clinical applications.

## 2 Theories on the vigilance decrement

Although the tasks and contexts wherein the vigilance decrement is observed are generally not very eventful (as discussed in relation to the *intensity* component in the prior section), it is actually quite challenging to maintain adequate performance over time. This is evidenced by the above-outlined real-life consequences of the vigilance decrement. While early research on vigilance took a largely empirical approach, aiming to capture and quantify the vigilance decrement as it was observed in real-life settings (Mackworth, 1948), it was only about two decades later that theoretical frameworks began to emerge to explain the phenomenon. These frameworks have taken different, sometimes opposing, forms, while other more integrative approaches have surfaced more recently.

### 2.1 Overload theories: resource-depletion account

Overload theories posit that the combination of a sparse display with a highly demanding discrimination task may be a source of stress (Dillard et al., 2019; Hancock and Warm, 1989; Szalma et al., 2004; Warm et al., 2008). This high demand would soon give rise to the exhaustion of available and limited cognitive resources that cannot be easily replenished, explaining the appearance of the attentional lapses that constitute the vigilance decrement (Grier et al., 2003; Warm et al., 2008). This theory has been tested showing that with increasing task demands, a greater vigilance decrement is observed (Epling et al., 2016; Head and Helton, 2014; Smit et al., 2004). Furthermore, this effect seems to be aggravated by sleep deprivation (Chua et al., 2017), where available resources would already be diminished.

### 2.2 Underload theories: mindlessness and mind-wandering accounts

While overload theories focus on the depletion of cognitive resources under high-demand conditions, underload theories offer an alternative perspective, positing that the monotonous nature of vigilance tasks leads to boredom (Danckert and Merrifield, 2018; Yakobi et al., 2021), which enables a gradual withdrawal from active or engaged task execution, toward a mindless execution of the task (Manly, 1999; Robertson et al., 1997). Furthering this idea, mind-wandering accounts pose that the attention that is withdrawn from the task does not merely vanish, but that its focus is actually directed toward internal thought, i.e., mind-wandering (Smallwood and Schooler, 2006). This process can be conceptualized as a shift from an exploitative state, where cognitive resources are allocated to task-relevant processing following established rules and goals, toward an explorative state characterized by internally directed cognition and reduced task engagement (Aston-Jones and Cohen, 2005; Kucyi et al., 2016; Mittner et al., 2016). Such a shift may reflect adaptive changes in attentional allocation during monotonous tasks, allowing the mind to seek alternative internal stimuli when external demands are insufficient to sustain engagement.

Underload theories have been supported by finding worse performance in less demanding tasks compared to dual tasks (Ariga and Lleras, 2011), as well as an increase in self-reported off-task states as the task progressed (Cunningham et al., 2000). Furthermore, when tasks were made more engaging or more variable (imposing a higher cognitive demand), improved performance was observed (Pop et al., 2012; Stearman and Durso, 2016; Thomson et al., 2015b).

### 2.3 Integrative approaches

Several theories have provided attentional insight that could potentially integrate the contradictory ideas and findings associated with under- and overload theories.

#### 2.3.1 Underload and overload as part of a continuum

Several accounts integrate both underload and overload across a continuum, wherein a middle ground for optimal performance can be achieved. These accounts often explain that vigilance performance depends on the degree of arousal (Esterman and Rothlein, 2019) or cognitive load (McWilliams and Ward, 2021), following the reverse U-shaped function that Yerkes and Dodson (1908) used to relate stress and cognitive performance. In this regard, underload would lead to what is coined as *passive fatigue*, whereas overload would lead to *active fatigue* (McWilliams and Ward, 2021; Saxby et al., 2013). A recent study has observed that tasks with both low and high cognitive load led to a pronounced vigilance decrement, whereas performance did not decay during a task with an intermediate cognitive load (Luna et al., 2022a), although this effect is not always replicated (Hemmerich et al., 2024).

#### 2.3.2 Dynamic resource allocation: the resource-control account

Thomson et al. (2015a) highlighted gaps within the underload and overload theories, proposing the resource control account. This model operates on the notion that: (i) the amount of cognitive resources we have available is constant (i.e., resources are not depleted as time progresses), (ii) the default state of the mind is mind-wandering, and (iii) with time-on-task our ability to exert executive control in order to maintain attention focused on the task at hand decreases. This decline in executive control would progressively hamper the ability to allocate mental resources toward the task at hand, as they gradually shift to support other task-unrelated thoughts, i.e., mind-wandering (Cunningham et al., 2000; McVay and Kane, 2012; Thomson et al., 2014), leading to the observed vigilance decrement in the task. While this theory has received some initial empirical support demonstrating the role of declining executive control as an explanation for the vigilance decrement (Luna et al., 2022b), it has not been extensively tested. Furthermore, the model posits that executive control decreases with time-on-task, not due to a lack of resources—since these are assumed to remain constant—but rather due to the adoption of less effortful processing strategies. This shift is believed to occur as participants adjust their performance to the low probability of encountering the critical target (Thomson et al., 2015a). However, this explanation for the decline in executive control remains somewhat abstract and theoretical. The following two models provide a more detailed examination of potential factors driving the adoption of different processing strategies as time-on-task progresses.

#### 2.3.3 Opportunity-cost model or cost-benefit models

Another relevant model that also considers that individuals flexibly adapt their performance during a task is the opportunity-cost model. While it has been defined more broadly for overall cognitive control (Kurzban, 2016; Kurzban et al., 2013), it can add an additional relevant perspective to explaining the vigilance decrement. The opportunity-cost model also considers that we operate with a limited but constant set of cognitive resources. However, with the ongoing performance of a task, we unconsciously (i.e., without these evaluations necessarily raising to awareness) weigh the benefit of continuing with this performance against the cost of losing the opportunity to perform other, potentially more rewarding or engaging tasks (Kurzban et al., 2013). The relevance of this model lies in the fact that the vigilance decrement can be considered not merely in terms of the loss of an ability, expended resources, or loss of sensitivity, but rather as a process that is tied in a more complex manner to emotional and motivational factors (Kurzban, 2016). Boksem and Tops (2008) offer an interesting brain-based account of this cost-benefit model explaining performance declines, expanding the role of dopamine beyond reactivity to reward into a basic component for motivation-guided behavior. Furthermore, dynamic shifts in performance driven by motivational factors can also be tied in with Aston-Jones and Cohen's (2005) Adaptive Gain Theory, which proposes that shifts in task engagement are regulated by changes in tonic and phasic norepinephrine activity. From this perspective, the vigilance decrement may reflect a neurobiologically-driven shift from task-focused exploitation to exploratory behavior when the perceived utility of sustaining performance on the same task declines.

#### 2.3.4 Decision making with an energy budget: the role of glycogen reserves

Many of the reviewed theoretical models that refer to “cognitive resources,” be it in the context of overload or allocation, often treat them as a fairly abstract concept (Grier et al., 2003; Thomson et al., 2015a; Warm et al., 2008). Christie and Schrater (2015) posit that a decline in cognitive performance with time-on-task can be accounted for by a depletion of the amount of glucose that is available to active neurons. However, they argue that cognitive performance is not solely dictated by raw resource availability. Instead, performance fluctuations can also reflect strategic allocation of metabolic resources based on reward contingencies. A key mechanism proposed in their model is the role of glycogen stored in astrocytes. Glycogen could act as a metabolic buffer, mobilized when tasks demand short bursts of elevated neural activity beyond the steady-state glucose supply. This allows for momentary performance recovery, even after initial depletion (Christie and Schrater, 2015). This offers an interesting integration of resource-depletion models and cost-benefit models, as the 2-fold expenditure of resources would explain different behavioral patterns based on cost-benefit analysis performed by the individual (i.e., tapping into glycogen stores to ensure continued performance, only if remaining in the current task is deemed a greater benefit or opportunity than switching to a different task). This could potentially explain the reports of null effects of hypoglycemia on sustained attention tasks (McAulay et al., 2001), if glycogen reserves are factored in as a putative compensatory mechanism. On the other hand, other accounts posit that declines in performance, and thus, indirectly the vigilance decrement, could be explained in terms of a “protective” neural mechanism against the potential damage of exerting extended high control over extended periods, which is experienced as cognitive effort (Holroyd, 2024). In this view, cognitive effort is tied not only to resource use but to mechanisms that limit prolonged high activation to preserve long-term neural health. Overall, while the idea of energy-budget-based decision making adds depth to models of vigilance, more empirical work is needed to determine whether glucose and glycogen dynamics could play a causal role in the vigilance decrement.

## 3 A closer look at the vigilance decrement: executive and arousal vigilance components

Further refining the above-outlined working definition of vigilance, a recent theoretical dissociation between two different types of vigilance has emerged. Luna et al. (2018) identified two distinct components that can be measured independently at the same time: an executive component and an arousal component. While both components of vigilance fit within the general definition of vigilance that was provided in Section 1.2, the distinguishing element between the two would be that of *control* (see Figure 1A), whereas the executive component requires a higher degree of decision making to gauge whether the critical stimulus is present and thus whether a response should be emitted or not, the arousal component requires less control, given that once the response to the critical stimulus is learnt, responses are provided in a more automatic and reactive manner.

More specifically, *executive vigilance (EV)* refers to the ability to monitor the environment to detect infrequent but critical signals, requiring higher-order cognitive processing as it encompasses monitoring the environment, accessing and updating working memory, making decisions, and executing accurate responses to the detected targets whilst inhibiting responses to non-targets according to task goals. This component can be observed in computerized tasks such as the above-mentioned MCT (Lichstein et al., 2000), the Sustained Attention to Response Task (SART; Manly and Robertson, 2005), or the Continuous Performance Test (CPT; Conners, 2000). In these tasks, participants are instructed to not respond to a frequently presented stimulus and respond only to a much less frequently presented target. Each trial, thus, requires one to evaluate whether one is presented with a target or not and emit the appropriate response. Within these tasks, the EV decrement is observed as the diminished ability to detect infrequent targets (i.e., hit rate with time-on-task; Luna et al., 2021a, 2018; Thomson et al., 2016).

On the other hand, *arousal vigilance (AV)* refers to the ability to maintain a fast response to an intermittent stimulus that always requires a response. As less deliberation and, thus, top-down control is required, correct responses can be emitted in a more general and automatic manner (Luna et al., 2018). This component can be measured with a computerized task such as the Psychomotor Vigilance Test (PVT, Lim and Dinges, 2008), where a countdown appears in the center of the screen at varying intervals, and it has to be stopped as fast as possible without executing a specific response (e.g., by pressing any available key from a keyboard). In this context, the AV decrement would be evidenced as an increment of reaction times (RT) and their variability (e.g., standard deviation of RT) with time (Lim and Dinges, 2008; Luna et al., 2021a, 2018).

A recently developed behavioral task which has been designed to be applied both in the lab and at home, the Attention Networks Test for Interactions and Vigilance—executive and arousal components (ANTI-Vea) allows to assess the functioning of the two dissociated vigilance components, as well as the main effects and interactions of the three attentional networks (i.e., phasic alertness, orienting, and executive control) (Coll-Martín et al., 2023; Luna et al., 2021a, 2018). The core task (completed in 60% of trials) is a standard flanker task, including alerting and orienting cues to measure the three attentional networks. The EV component is measured through the detection of an infrequent (in 20% of trials) large vertical displacement of the central target of the arrow flanker task, respective to the flankers, and the AV component is measured in a sub-task more similar to the PVT, where infrequently (in 20% of trials) a countdown appearing on the screen has to be stopped as fast as possible, without a specific response mapping (i.e., correct responses constitute pressing any key on the keyboard).

This independent and simultaneous assessment of vigilance components may help reconcile contradictory findings—particularly when considering data beyond the behavioral responses. In fact, some dissociations of the two components of vigilance have already been observed at the physiological level, through caffeine consumption and physical exercise (Sanchis et al., 2020; Sanchis-Navarro et al., 2024), as well as the neural level, evidenced by differing electrophysiological profiles (Luna et al., 2023) and responses to the application of non-invasive brain stimulation (NIBS, Hemmerich et al., 2023, 2024; Luna et al., 2020). Future research exploring distinct neural correlates or active manipulations of physiological activity could further clarify the dissociation between these components, extending beyond their conceptual importance.

## 4 The malleability of the vigilance decrement: modulating factors

**Figure 2:**
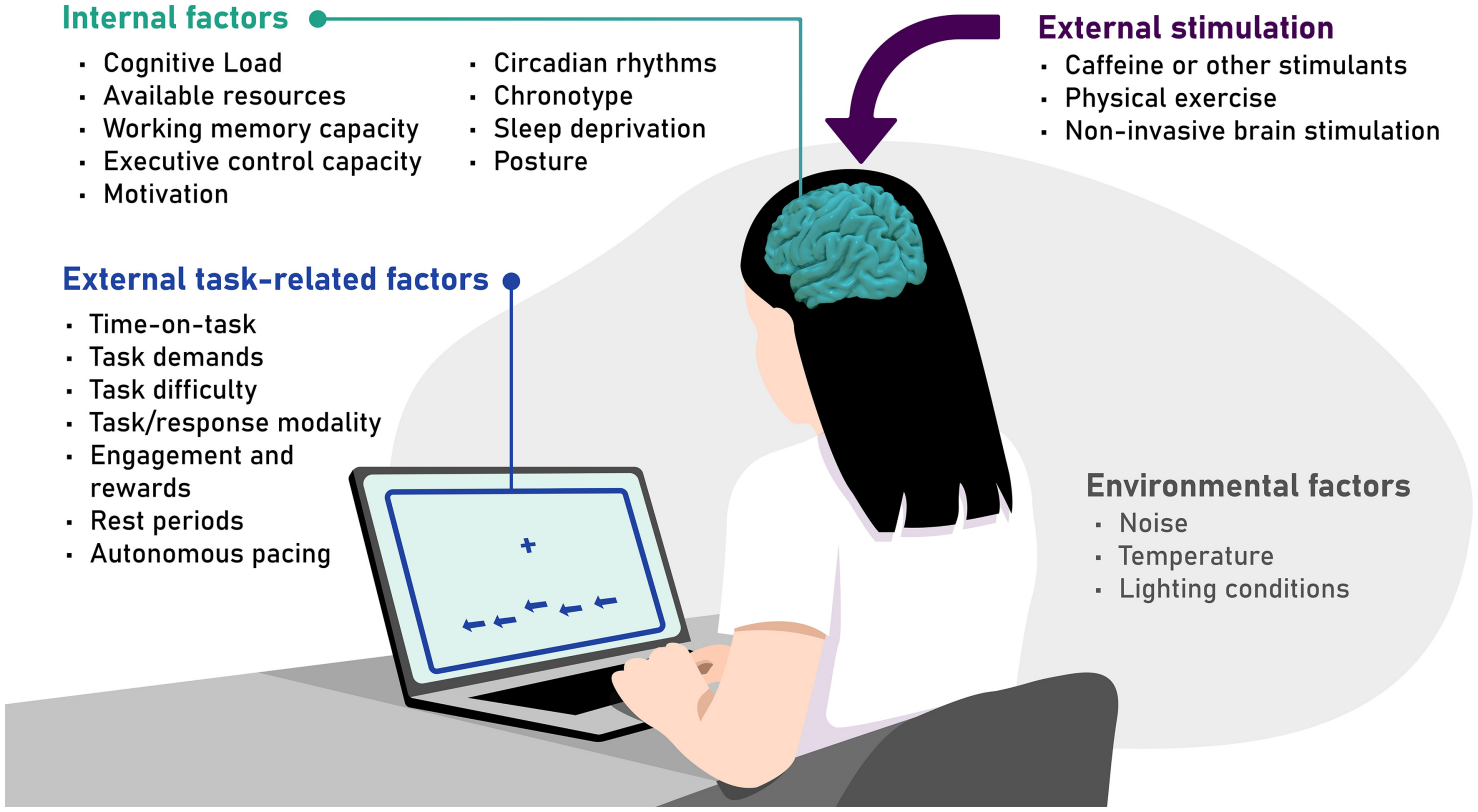
A comprehensive yet not exhaustive overview of relevant factors that can modulate the vigilance decrement, grouped into external task-related factors, internal factors, environmental factors, and external stimulation.

The theories outlined in the previous section suggest that the vigilance decrement is a multifaceted phenomenon that can be influenced by a wide range of factors. A representative, though not exhaustive, list of relevant factors is detailed below, grouped into external task-related factors, internal factors, environmental factors, and the application of external stimulation (see Figure 2 for an overview).

### 4.1 External task-related factors

Time-on-task can be considered a crucial contributor and an inherent property of the vigilance decrement (Warm et al., 2008). Nevertheless, it must be considered that a decrement of vigilance with time-on-task is not always observed (Epling et al., 2019). It has been argued that a time-on-task-dependent vigilance decrement may be more evident at the group level, but more difficult to grasp at an individual level (Parasuraman and Jiang, 2012). This could be partially influenced by the different factors outlined in this section.

Precisely, task demands and task difficulty greatly shape the vigilance decrement. There's evidence for worse performance under high demands (Epling et al., 2016; Head and Helton, 2014; Smit et al., 2004) explained by the resource overload theory, as well as evidence for worsened performance under low demands (Ariga and Lleras, 2011) explained by underload theories. Whilst reverse-U-shaped patterns have been observed, with both low and high demands producing a vigilance decrement, it can be reduced with intermediate demands (Luna et al., 2022a). Furthermore, greater vigilance decrements have been observed with increased perceptual difficulty (i.e., when target stimuli are less salient or detectable) (Ballard, 1996; Helton et al., 2010). On the contrary, task difficulty induced by increasing targets' perceptual variability has led to better performance (Thomson et al., 2015b). This may tie in with subjective perceptions of task engagement (see Section 4.2), as a potential explanation of these diverging results. As a case in point, increasing task engagement or providing rewards may influence vigilance performance. Additional steps or processing demands can improve the engagement of the task, facilitating performance (Pop et al., 2012; Thomson et al., 2015b). Additionally, incorporating rewards into the vigil period has shown to improve performance, albeit only for a brief burst (Reteig et al., 2019).

Some authors have also highlighted the importance of incorporating rest periods into a vigil, as to restore or partially restore vigilance (Arrabito et al., 2015; Helton and Russell, 2017; Helton and Wen, 2023). Furthermore, it has been shown that autonomous pacing (i.e., having control over the pace of stimulus presentation) in a vigilance task can benefit performance (Scerbo et al., 1993). Lastly, broader factors such as the task modality could also influence the vigilance decrement. While visual targets are the most commonly used modality, the vigilance decrement can also be observed with auditory (Szalma et al., 2004) and vibrotactile targets (DeLucia and Greenlee, 2022); with auditory—compared to visual—stimuli posing an advantage on vigilance performance (Szalma et al., 2004).

### 4.2 Internal factors

On top of objective manipulations of cognitive demand (as discussed in the prior section), individuals may differ on their thresholds for what might be considered high or low cognitive load (Vergallito et al., 2018), which might be especially relevant in clinical contexts or during development and aging (Ballard, 1996). Additionally, available resources may vary and influence vigilance performance. As pointed out in Section 2.3.4, resources are often used in an abstract manner. Direct measures of metabolic consumption suggest different potential resource storages that can be accessed; influenced by time-on-task, demand, or incentives (Christie and Schrater, 2015).

Furthermore, working memory load seems to affect the vigilance decrement when the overload occurs in the same modality in which the vigilance decrement is being measured, but not across modalities (Caggiano and Parasuraman, 2004). However, other studies find no effect of working memory load on the vigilance decrement within the same modality (Martínez-Pérez et al., 2023). Executive function capacity may also influence vigilance performance. According to Thomson et al. (2015a), the dwindling of executive control impedes the correct allocation of resources to a task, leading to the vigilance decrement. While, interestingly, one study observed no decrement of executive control with time-on-task (Zholdassova et al., 2021), other studies report a correlation between EV performance (overall hits in EV trials) and overall errors in executive control (Luna et al., 2021c), as well as a significant albeit relatively small correlation between the EV decrement and the decrement in cognitive control across time-on-task (Luna et al., 2022b).

Intrinsic motivation may also play an important role in the vigilance decrement. In fact, Hancock (2013) proposes that the vigilance decrement stems from the external imposition of the vigil. Furthermore, it should be noted that laboratory tasks are detached from the consequences that arise from the vigilance decrement in real-life scenarios, which can impact the motivation to perform at a certain standard or facilitate operating at a pace that adapts to individual and momentary needs. Having to detect signs pointing toward signs of cancer when inspecting a mammography, for example, produces much higher stakes than a laboratory task, where not detecting the critical stimulus has virtually no consequences. In fact, in the prior example, no vigilance decrement was observed, as sensitivity to detect the critical signal did not decrease with time (Taylor-Phillips et al., 2015).

More general states of the organism may further influence cognitive performance (including vigilance). Vigilance may fluctuate across the day in line with circadian rhythms (Valdez, 2019), and can be further affected by performing outside of the optimal time window determined by chronotype, especially for evening types (Martínez-Pérez et al., 2020) or when attentional deficits such as attention deficit hyperactivity disorder (ADHD) are present (Gabay et al., 2022). The vigilance decrement is also exacerbated by sleep deprivation (Hudson et al., 2020), especially when task demands are higher (Chua et al., 2017). Lastly, posture may also affect performance, with some results showing that prolonged standing has shown to slow down responses in a vigilance task to keep the same level of accuracy (Baker et al., 2018). On the other extreme, lying down, as compared to sitting or standing, has been associated with increased mind-wandering and worse cognitive performance (Yang et al., 2022).

### 4.3 Environmental factors

The environment in which a vigilance task is performed may also impact vigilance performance. For example, noise has shown to affect the vigilance decrement in a variable way, and it is suggested that it may interact with other factors such as task demands (Ballard, 1996; Hancock and Warm, 1989). While a constant and predictable noise may in fact increase task engagement, and thus, vigilance performance (Helton et al., 2009), less predictable noises may impair performance (Carter and Beh, 1987). Furthermore, deviations from an intermediate temperature into either extreme seem to negatively affect vigilance performance (Ballard, 1996). Lastly, higher light temperatures (i.e., blue light) have been associated with better vigilance performance (Chellappa et al., 2011), although this effect, together with an impact of light intensity is not always observed (Souman et al., 2018).

Beyond specific environmental variables, a person's surroundings as a whole may also influence vigilance performance and shape the time-course of the vigilance decrement. This might become evident when comparing participants' performance of an in-lab vigilance task—where environmental parameters are highly controlled or systematically manipulated—with an online administration of the same task—where these external factors are less controlled and expected to be more heterogeneous. Interestingly, recent studies have shown that actually no substantial differences are observed between in-lab and online administrations of vigilance tasks, tested on an EV task (Claypoole et al., 2018; Thomson et al., 2016) and in a task testing both EV and AV (Luna et al., 2021c). However, Claypoole et al. (2018) argue that the type of vigilance task, the length of the task, and the metrics used to evaluate the vigilance decrement should be important factors to consider replication of results in less controlled environments, such as online applications of vigilance tasks.

Given the inconclusive evidence regarding the impact of environmental factors, it remains advisable to minimize and standardize contextual influences as best as possible to enhance the reliability of vigilance performance.

### 4.4 External stimulation (or countermeasures)

External stimulation of the organism, that can directly or indirectly affect the brain can also impact vigilance performance. For example, Sanchis et al. (2020) observed improved AV performance with caffeine intake. Beneficial effects of caffeine administration have also been reported for sustained attention, whereas methylphenidate reduced self-reported fatigue (Repantis et al., 2021). Furthermore, physical exercise at moderate intensity has shown to mitigate the EV decrement (Sanchis et al., 2020). When directly comparing the effect of exercise intensity on attentional and vigilance performance, beneficial effects on EV performance were only observed in a light-intensity as compared to a vigorous condition or a baseline physiological state; without any effects on AV (Sanchis-Navarro et al., 2024).

NIBS techniques have been increasingly explored as potential countermeasures to the vigilance decrement. Among the different stimulation techniques, transcranial direct current stimulation (tDCS) has been most frequently used. For tasks assessing EV, a substantial number of studies report no effect of tDCS on vigilance performance, across the use of anodal tDCS over left frontal (Coulborn and Fernández-Espejo, 2022; Dai et al., 2022; Filmer et al., 2019; Hussey et al., 2020; Martínez-Pérez et al., 2023), central (Adelhöfer et al., 2019), parietal (Coulborn et al., 2020), and cerebellar regions (Erdogan et al., 2023). Yet, several other studies have found beneficial effects of tDCS on EV, such as increased hit rate with bilateral frontal tDCS at opposing polarities (Nelson et al., 2014), increased accuracy with anodal tDCS over the lDLPFC (McIntire et al., 2014) and both anodal and cathodal tDCS over the left frontal eye fields (lFEF; Nelson et al., 2015), improved sensitivity with anodal tDCS over the lFEF (Gan et al., 2022), and a reduction in lapses with anodal tDCS over right frontal regions (Brosnan et al., 2018). Notably, multicomponent tasks assessing both EV and AV (e.g., the ANTI-Vea) have shown beneficial effects specifically in the EV component when anodal tDCS was applied over the right posterior regions (Hemmerich et al., 2023; Luna et al., 2020), suggesting that this vigilance domain may be more responsive to tDCS. It has been further observed that these beneficial effects hold up only under conditions of high cognitive demand (Hemmerich et al., 2024). Lastly, studies targeting only AV have produced fewer and more cautious conclusions. While one tDCS study reported no effects on AV performance through anodal tDCS over the lDPLFC (Borragán et al., 2018), another one observed beneficial effects on RT with bilateral frontal anodal tDCS (Alfonsi et al., 2023). Notably, most of these studies employed conventional tDCS procedures, which use two larger stimulation sites of the opposite polarity, as opposed to HD-tDCS, which permits more focal and pseudo-unipolar stimulation with a central electrode surrounded by return electrodes of the opposite polarity (Alam et al., 2016; Edwards et al., 2013), which was only used in a minority of the cited studies (Hemmerich et al., 2023, 2024; Luna et al., 2020; Martínez-Pérez et al., 2023).

Transcranial alternating current stimulation (tACS) has also been applied, though studies are sparser. Both theta-tACS and alpha-tACS over right frontal brain regions have shown to mitigate the AV decrement, whereas only alpha-tACS, but not theta-tACS, mitigated the EV decrement (Martínez-Pérez et al., 2022). It must be noted that these results were only obtained when participants performed outside of the optimal time-window during the day, as determined by their chronotype. Furthermore, Kasten et al. (2016) observed no effects on EV with alpha-tACS targeting central parieto-occipital brain region; while Wei et al. (2021) only observed effects on AV performance in a post-stimulation period. Early evidence also suggests that transcranial random noise stimulation (tRNS) may positively influence EV. Harty and Cohen Kadosh (2019) reported enhanced EV performance, particularly at lower tRNS intensities targeting frontal and parietal brain regions. By contrast, transcutaneous auricular vagus nerve stimulation (taVNS) has thus far not yielded significant benefits in either vigilance component. Two studies, one examining both AV and EV (Luna et al., 2025) and another examining only AV (Zhao et al., 2023), reported null findings, despite the hypothesized role of the vagus nerve in modulating arousal and attentional functions. Finally, repetitive transcranial magnetic stimulation (rTMS) has shown to lead to reduced errors and faster RT in tasks assessing EV when targeting the lDLPFC (Kim et al., 2020) and improved AV performance when targeting the right middle frontal gyrus (Zhu et al., 2024).

NIBS might be a promising tool to counteract the decrement of vigilance. However, outcomes vary considerably depending on the type of stimulation, targeted neural region, and the component of vigilance under assessment, underlining the need for future research in this area.

The decline in vigilance performance likely results from a complex interplay of external, internal, and environmental factors such as depletion of cognitive resources or executive control, changes in arousal levels, task characteristics, and individual strategies for managing attention and workload. Understanding this interplay is crucial for further developing effective interventions to mitigate the vigilance decrement. As research continues, a more comprehensive model integrating these various aspects may emerge, offering a deeper understanding of vigilance and its decrement. On the other hand, it must be noted that this is not an exhaustive list of all potential factors that may modulate vigilance functioning and the evidence of some of them may, in some cases, originate from studies with smaller samples that are less generalizable. For now, this list serves to underline the importance of adequately controlling and reporting these factors when conducting vigilance research.

## 5 Neural correlates of the vigilance decrement

Neuroimaging techniques can provide a better understanding of what occurs in the brain when vigilance is exerted and when it decays over time. This may be achieved by, on the one hand, inspecting more stationary cortical and subcortical regions, or networks of regions, which either exhibit fluctuations in activation during vigilance tasks or in response to specific task manipulations, or with the association of characteristics of anatomical structures with vigilance performance. On the other hand, neuroimaging techniques with a higher temporal resolution offer insight into more dynamic correlates, associating neural oscillations with vigilance performance and with the vigilance decrement over time.

### 5.1 Stationary vigilance “hubs” and networks: evidence from functional and structural neuroimaging

#### 5.1.1 Functional neuroimaging

**Figure 3:**
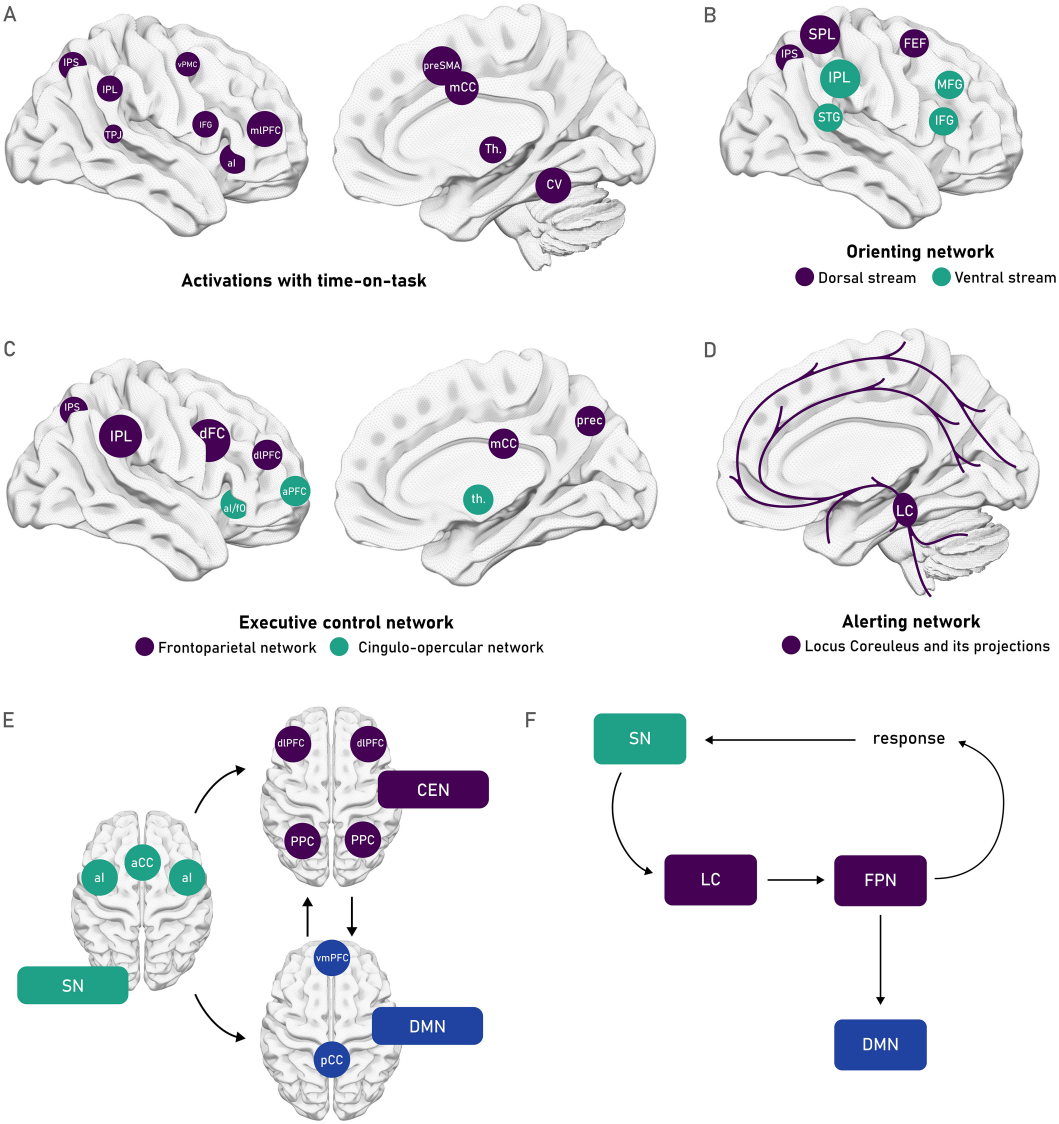
Schematic depiction of different neural networks relevant for attention and vigilance **(A)** Foci of brain activity that showed a greater activation with task duration identified within a general network of areas activated during vigilant attention in the coordinate-based meta-analysis performed by Langner and Eickhoff (2013). The right-lateralized set of areas obtained included the anterior insula, presupplementary motor area (pre-SMA), midcingulate cortex (mCC), midlateral prefrontal cortex (mlPFC), ventral premotor cortex (vPMC), inferior frontal gyrus (IFG), inferior parietal sulcus (IPS), and adjacent inferior parietal lobule (IPL), temporoparietal junction (TPJ), thalamus, and cerebellar vermis. **(B)** Posner and Petersen's (1990) orienting network that can be subdivided as characterized by Corbetta and Shulman (2002) into the dorsal top-down stream (depicted in purple), composed of the frontal eye fields (FEF) as well as the IPS and superior parietal lobe (SPL); and the ventral bottom-up stream (depicted in green) composed of the temporoparietal junction (TPJ) and the ventral frontal cortex (VFC). **(C)** The executive control network identified by Posner and Petersen's (1990), spans the networks that Dosenbach et al. (2007, 2008) further distinguished into the frontoparietal network (in purple) composed of the IPS, IPL, dorsal frontal cortex (dFC), and dorsolateral prefrontal cortex (dlPLFC); and the cingulo-opercular network (in green), composed of the anterior insula/frontal operculum (aI/fO), and the anterior prefrontal cortex (aPFC). **(D)** The alerting network (Posner and Petersen, 1990) that is controlled by the release of norepinephrine from the cortical projections of the Locus Coeruleus. **(E)** The cingulo-opercular system has also been conceptualized as the salience network [SN, composed of the aI, and the anterior cingulate cortex (aAC)], which acts as a relevant relay point between the central executive networks [CEN, composed of the dlPFC and posterior parietal cortex (PPC)], and the default mode network [DMN, composed of the ventromedial PFC (vmPFC), and the posterior cingulate cortex (pCC)] as proposed by Menon and Uddin (2010). **(F)** The SN has further been proposed to aid in the inhibition of the DMN by the FPN, driven by recruitment of the projections of the LC (Unsworth and Robison, 2017).

Given the above-outlined overlap of vigilance with other attentional functions and its interaction with other cognitive processes, it is to be expected that it cannot be circumscribed to one specific neural location. In fact, it has been established by a coordinate-based meta-analysis on functional Magnetic Resonance Imaging (fMRI) and Positron Emission Tomography (PET) data that vigilance is related to neural activity distributed across different neural networks or clusters, many of which are lateralized toward the right hemisphere (Langner and Eickhoff, 2013). While Langner and Eickhoff (2013) considered a noticeably low duration criterion (>10 s into the task) to include studies, within the aforementioned identified areas, a further right-lateralization was observed when looking at foci of brain activity correlating with longer task durations (see Figure 3A). In line with these results, the right-lateralization of vigilance has also been reported from lesion studies. Patients who had suffered a lesion to right frontal regions, presented a larger vigilance decrement than patients with left frontal or other lesion sites (Koski and Petrides, 2001; Molenberghs et al., 2009; Rueckert and Grafman, 1996). A more recent study has additionally shown that patients with lesions to the right-hemisphere also present steeper within-block vigilance decrements compared to healthy controls (Brosnan et al., 2022). Further evidence of this lateralization has also been gathered from neuroimaging studies with healthy participants. An earlier study showed that right frontal and parietal areas show activation during vigilance tasks in PET imaging (Pardo et al., 1991). On the other hand, perfusion fMRI data has shown that blood flow in the frontoparietal network is reduced from pre- to post-task, and this reduction in blood flow was associated with a vigilance decrement (Lim et al., 2010). In line with this right-lateralization of vigilance, several studies report improved vigilance with tDCS applied over the right frontal cortex (Brosnan et al., 2018), as well as both tDCS (Luna et al., 2020) and tRNS (Harty and Cohen Kadosh, 2019) applied over right frontoparietal regions. In contrast, absent effects on vigilance have been frequently reported with anodal tDCS protocols over left frontal regions (Coulborn and Fernández-Espejo, 2022; Dai et al., 2022; Filmer et al., 2019; Hussey et al., 2020; Martínez-Pérez et al., 2023). However, some beneficial effects have also been reported with anodal tDCS over left frontal regions (Gan et al., 2022; McIntire et al., 2014), and also with bilateral targeting of right and left frontal regions with opposing polarities (Nelson et al., 2015, 2014). These findings support the dominant role of right-hemisphere structures in vigilance but suggest that left-hemisphere involvement, possibly through compensatory mechanisms, should not be overlooked. It is possible that the vigilance decrement also reflects an unbalanced inter-hemispheric synchronization, which may be mitigated by external stimulation that helps restore this balance.

The regions identified by Langner and Eickhoff (2013) show an overlap with networks identified in other attentional models, such as the *dorsal top-down stream* and the *ventral bottom-up stream* identified by Corbetta and Shulman (Corbetta et al., 2008; Corbetta and Shulman, 2002). These two streams make up the orienting network identified by Posner and Petersen (1990) as depicted in Figure 3B, which regulates goal- and stimulus-driven allocation of attentional resources to relevant stimuli (Petersen and Posner, 2012; Posner and Petersen, 1990). Furthermore, some overlap can also be observed with the executive control network (Petersen and Posner, 2012; Posner and Petersen, 1990), which encompasses what Dosenbach et al. (2007, 2008) characterized as a *frontoparietal network*, associated with initiating and adjusting control over ongoing performance; and the *cingulo-opercular network*, associated with a stable maintenance of task-goals across longer time periods (see Figure 3C). As depicted in Figure 3D, most of these regions are reached by the alerting network, composed of the cortical projections of the LC. Further potential contributions for adequate vigilance functioning can be found in relation again to the cingulo-opercular network, this time identified as part of the salience network (SN), which has been proposed to assist in balancing exogenous or task-driven activity in the central executive network (CEN) and the more endogenous or self-referential activity of the default mode network (DMN), as shown in Figure 3E (Menon, 2011; Menon and Uddin, 2010). Achieving this balance could play a key role in regulating neural activity for task-related thoughts and neural activity for self-referential thoughts or mind-wandering. Lastly, in a more directional model, Unsworth and Robison (2017) propose that the inhibitory effect of the frontoparietal network (FPN) on the DMN is aided by the SN, driven by the projections of the LC, as shown in Figure 3F. However, in relation to this last model, a recent study using taVNS, showed effects on the LC norepinephrine system with active stimulation, but no effects on vigilance performance (Luna et al., 2025).

In line with the prior established roles of attentional networks and the DMN, it must be noted that the notion of the DMN as task-negative, or the attribution of its activity with degraded performance has been challenged by findings from Esterman et al. (2013), indicating that instead, a push-pull relationship between the DMN and the dorsal attention network (DAN) subserves different attentional states. An “in the zone,” more stable and automatic processing that can arise in less challenging tasks, is characterized by higher DMN activity, and permits less effortful processing at the expense of risking errors if DMN activity increases past a certain threshold. During more demanding tasks, a more effortful “out of the zone” state emerges, characterized by higher activity in the DAN, where errors are more likely to occur if insufficient control is exerted by the DAN (Esterman et al., 2013).

While sustaining the idea that there is no unique location that subserves vigilance, the right posterior parietal cortex (rPPC) may play a fundamental role in permitting adequate vigilance performance. The rPPC—integrated by the superior parietal lobule (SPL) and the inferior parietal lobule (IPL)—plays a crucial role in spatial attention, given that the IPL is the main lesioned area in hemispatial neglect (Malhotra et al., 2009; Molenberghs et al., 2009). However, neglect patients often present additional deficits in vigilance/sustained attention (Malhotra et al., 2009), which highlights the involvement of this region in vigilance functioning. Furthermore, the rPPC shows a heightened hemodynamic response to the presentation of infrequent (Stevens et al., 2005) and novel (internal and external) stimuli (Singh-Curry and Husain, 2009). Additionally, it has also been associated with the active maintenance of task goals (Singh-Curry and Husain, 2009). This has led some authors to establish the rPPC as a “convergence node” between the ventral attention network and the DMN: thus considering its relevant role in maintaining task goals active, whilst flexibly reacting toward novel or salient stimuli and relaying between task-relevant and task-irrelevant regions (Giacometti Giordani et al., 2023). This role can be feasible on a structural level due to the densely interconnected core that has been observed in this region, with further dense connections to other neural regions (Hagmann et al., 2008). Conceptually, the rPPC could play a relevant role as a relay switch in the complex interplay of forces that lead to the vigilance decrement: resources, mind-wandering, executive control, motivation, and cost-benefit analyses. Further support for the potential involvement of the rPPC in vigilance, specifically executive vigilance, comes from findings of a mitigated EV decrement when this area was targeted with tDCS, showing promising behavioral and neurophysiological effects (Hemmerich et al., 2023; Luna et al., 2020).

As a counterpoint, some accounts suggest that the right-lateralization of vigilance is observed only in simpler, less demanding tasks, whereas in more complex tasks a bilateral hemispheric activation is observed (Helton et al., 2010). This observation highlights the fact that despite the above-discussed relevance of the rPPC for vigilance, the importance of broad networks in supporting the adequate functioning of vigilance must be considered. In line with this, Rosenberg et al. (2016) have established a connectome-based predictive model that can predict individual differences in sustained attention functioning from task-based as well as resting-state functional connectivity data. This model can predict attentional fluctuations within and between task blocks and sessions, as well as responsiveness to external modulations of attention, such as the administration of sedatives (Rosenberg et al., 2016). The model has also proven to effectively predict attention-deficit symptom severity in an independent sample (Rosenberg et al., 2020). Interestingly, this model includes regions beyond the canonical regions associated with attention (salience, frontoparietal, or default mode networks), and implicates other regions such as the cerebellum (Rosenberg et al., 2016). Although, as already mentioned, an intervention with tDCS targeting the cerebellum has not shown significant effects on vigilance functioning (Erdogan et al., 2023); which could suggest that these non-canonical regions have less principal roles, and participate more at the network level to promote adequate levels of vigilance.

#### 5.1.2 Structural neuroimaging

Diffusion Weighted Imaging (DWI) offers insight into structural anatomical features that are highly relevant for the adaptive signal transmission required by attentional processes, by, for example, linking the integrity of white matter tracts to attentional functioning. Considering the above-reviewed evidence, pathways connecting frontoparietal areas, such as the branches of the superior longitudinal fasciculus (SLF) could be of special interest (Thiebaut De Schotten et al., 2011). The SLF connects frontal, temporal, parietal, and occipital regions and has been subdivided into three branches: SLF I (dorsal), SLF II (medial), and SLF III (ventral) (Janelle et al., 2022). As a case in point, a higher fractional anisotropy (FA) in the SLF in typically developing children has been associated with better sustained attention performance (Klarborg et al., 2013). Moreover, adolescents with ADHD show a strong relationship between reported inattentive symptomatology and alterations in the right SLF (Chiang et al., 2015). Furthermore, this link has also been established in healthy adults, where higher fiber density (FD, an estimate of axon density) of the SLF I was associated with fewer attentional lapses during a global-local task (Clemente et al., 2021). Furthermore, Luna et al. (2021b) observed that higher white matter integrity of the SLF I in healthy adults was associated with faster response times for correct responses in an EV sub-task. However, no significant associations were observed with other more reliable or direct indicators of the vigilance decrement (such as the decrement of hits or sensitivity with time-on-task) (Luna et al., 2021b). Theoretically, the SLF I is thought to mediate goal-directed attentional processes within the dorsal attention network. This activity may be modulated by the SLF II, which acts as a communication bridge between the dorsal and ventral attention networks, enabling the redirection of attention toward salient stimuli identified by the SLF III (Thiebaut De Schotten et al., 2011).

Another tract that may be relevant for vigilance is the right cingulate fasciculus (or cingulum), which runs around the corpus callosum (Catani and Thiebaut de Schotten, 2008). Higher FA in the cingulum has been associated with higher sensitivity to infrequent targets in an EV-like task (CPT, Takahashi et al., 2010); which might be related to this structure's role as a mediator between the FPN and DMN, balancing task-related and self-referential processes (Menon, 2011). The dorsolateral prefrontal-caudate tract has been associated with vigilance performance (Chiang et al., 2015), while the right inferior fronto-occipital fasciculus (IFOF) may play a role in response inhibition (Pironti et al., 2014), potentially helping to suppress task-irrelevant stimuli during vigilance tasks. The IFOF may also exert fast top-down control from frontal regions over visual areas, supporting attentional modulation of perception (Bartolomeo and Seidel Malkinson, 2019). Additionally, considering the detrimental effect of sleep deprivation on the vigilance decrement (Lim and Dinges, 2008), it is worth noting that DWI data has also been used to predict individual vulnerability to sleep deprivation. Wang et al. (2022) found that the integrity of the SLF, posterior corona radiata, anterior limb of the internal capsule, as well as the body and genu of the corpus callosum, best predicted vulnerability to sleep deprivation, suggesting that inter-individual differences in white matter structure may underlie resilience to attentional decline in extremer conditions. Moreover, Niogi et al. (2010) identified positive correlations between white matter FA and the functioning of Posner and Petersen's (1990) three attentional networks: the alerting network was linked to the posterior limb of the internal capsule (PLIC), the orienting network to the splenium of the corpus callosum, and the executive control network to the left anterior corona radiata. These associations suggest a distributed anatomical basis for distinct attentional components.

Importantly, examining how the effects of NIBS spread across the brain may offer deeper insights into the white matter structures that support vigilance-related functions. By stimulating a specific cortical region that serves as a node within a broader network, it is possible to observe network-level effects at both neural and behavioral levels (Hartwigsen and Silvanto, 2023; Momi et al., 2021). As a case in point, stimulating nodes of the DMN with TMS has shown to produce wider effects within white-matter wiring of the DMN (Esposito et al., 2022; Momi et al., 2021). Similarly, by stimulating nodes of the FPN with TMS, the involvement of the SLF has been prominently related to broader attentional and cognitive functioning (Botta et al., 2021; Martín-Arévalo et al., 2019; Martín-Signes et al., 2019, 2021, 2024; Quentin et al., 2016). Furthermore, the successful modulation of vigilance via transcranial electrical stimulation over right frontal, parietal, and frontoparietal regions (Brosnan et al., 2018; Hemmerich et al., 2023; Luna et al., 2020) mentioned in prior sections, could also be linked to reaching nodes of the FPN that then distribute across the whole structure. The associations with vigilance and deeper-set white matter structures might be more difficult to establish, as fewer nodes are available to be reached via NIBS. Nonetheless, the fact that these different networks subserve vigilance functioning also permits thinking about network effects and the distribution of effects from brain stimulation at a more indirect level. However, the more direct links that have been established with TMS, in predicting stimulation outcomes, remain underexplored in applications specific to vigilance and in relation to other NIBS techniques.

### 5.2 Dynamic models of vigilance: the role of neural oscillations

**Figure 4:**
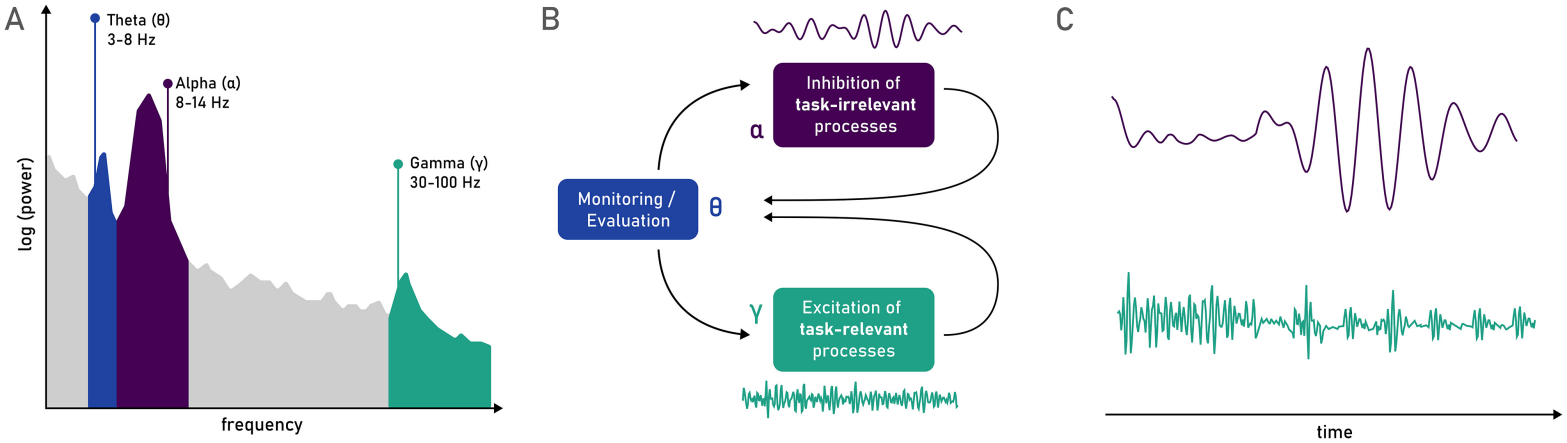
**(A)** Theta (θ), alpha (α), and gamma (γ) bands represented in a power density spectrum. **(B)** The oscillatory model of sustained attention proposed by Clayton et al. (2015), in which theta is responsible for supervising the attentional process (as proposed also by Fiebelkorn and Kastner, 2019), inhibiting task-irrelevant processes via oscillations in the alpha band, and re-energizing task-relevant processes via oscillations in the gamma band. **(C)** Gating of gamma oscillations by alpha oscillations (Osipova et al., 2008), that could constitute a relevant rhythmic purging of task-irrelevant information to sustain vigilance across time (Sadaghiani and Kleinschmidt, 2016).

Despite the monotonous nature and unchanging demands imposed by simple vigilance tasks, neural regions and networks associated with the adequate functioning of attention (and by extension vigilance) are still highly dynamic (Fiebelkorn and Kastner, 2019), especially when zooming in from the decrement at the minute/hour level, and instead inspecting finer-grained time-scales (i.e., seconds and milliseconds). This latter level of observation permits the use of finer time-windows to observe information-gating processes (Corriveau et al., 2025), which are fundamental for vigilance (Rosenberg, 2025). This characteristic can be grasped by associating vigilance with oscillations in specific frequency bands in studies inspecting electrophysiological (EEG) data, such as those illustrated in Figure 4A. Fiebelkorn and Kastner's (2019, 2020) rhythmic theory of attention posits that lower-frequency oscillations in attentional networks organize neural activity into rhythmically alternating states. During tasks requiring vigilance, this would lead to intermittent periods of lower perceptual sensitivity, during which, for example, an attended location is re-selected based on both stimulus properties and task goals (Fiebelkorn and Kastner, 2019). The rhythmic sampling is orchestrated by oscillations in the theta band (3–8 Hz) inherent to the FPN, which determines activity in higher frequency bands, influencing behavioral outcomes (Helfrich et al., 2018). In line with this, Reteig et al. (2019) observed an increment of the temporal variability in cortical responses, indexed through inter-trial phase clustering of theta, along with the expected decrement of performance with time-on-task. Thus, precise rhythmic stability may be required for stable vigilance performance, and its destabilization might be a putative origin of the vigilance decrement.

This orchestrating role of neural oscillations in the theta band has been integrated into a more complex model in order to explain sustained attention via the interplay of different neural oscillations, and may also serve to understand vigilance functioning. Stuss et al. (1995) presented a schematic proposal of how attention (and by extension vigilance) maintenance is orchestrated: a supervisory system must, on the one hand, reactivate target schemata that are necessary to detect the infrequent target stimulus, whilst on the other hand ensuring that other competing schemata do not capture behavior by inhibiting them. Lastly, this monitored information must return back to the control of the schemata to adjust responses accordingly (Stuss et al., 1995). Clayton et al. (2015) propose a very similar model, where specific oscillations are attributed to these different functions (see Figure 4B). The monitoring and evaluation of task performance in relation to task goals are associated with theta oscillations in frontomedial regions, and consequent frontomedial theta-band phase synchronization relays modulatory signals to low-level, sensorimotor areas. Oscillations in the gamma band (>30 Hz) are associated with the excitation of task-relevant processes, whilst oscillations in the alpha band (8–14 Hz) are associated with the inhibition of task-irrelevant processes or stimuli. Lastly, bidirectional communication across frontoposterior networks (i.e., the relegation of inhibition and excitation based on task-goals, as well as the return of feedback from actual task execution) is handled via low-frequency phase synchronization (Clayton et al., 2015).

Regarding the specific role of alpha oscillations in vigilance, many studies report an increment of alpha power with time-on-task (Benwell et al., 2019; Boksem et al., 2005; Compton et al., 2019; Hemmerich et al., 2023; Luna et al., 2020). Craig et al. (2012) specifically note that across reviewed studies observing time-on-task induced fatigue, the most commonly reported change in EEG was observed as an increment of activity in the theta and alpha bands. Another study observed increments of lower alpha power (7.5–10 Hz) with increased time and fatigue, especially in parietal electrodes; whilst other frequency bands showed no relationship to fatigue (Boksem et al., 2005). Replicating the increment of alpha power with time-on-task, Benwell et al. (2019) also observed a reduction in the peak frequency of alpha as time progressed. In a slightly different approach, during a driving task and an additional auditory vigilance task, Sonnleitner et al. (2014) observed an increment of both response times to brake in response to an on-road stimulus as well as the rate of alpha spindles [short bursts of alpha band activity, comprehended between 500 ms up to several minutes (Simon et al., 2011)] with time-on-task, which were exacerbated by the addition of a secondary task. It has been argued that in some conditions (such as driving situations), alpha spindles can more accurately capture fatigue than measures of alpha band power (Simon et al., 2011). The increment in alpha power with time-on-task is interpreted as: (i) indicating an attenuation in information processing over time (Pershin et al., 2023), or (ii) reflecting an increased effort to maintain vigilance, especially under conditions of higher demand, either due to externally imposed load or due individual differences such as older age, brain injury or sleep deprivation (Klimesch, 1999). On the other hand, alpha band activity has also been associated with mind-wandering. For example, Compton et al. (2019), observed that mind-wandering reports were positively associated with higher pre-stimulus alpha power. This has been further integrated with research that joins EEG and fMRI data recorded at rest. These concomitant recordings show a negative correlation between occipital alpha power and FPN activity (Mo et al., 2013). On the other hand, a positive correlation between occipital alpha power and BOLD activity in nodes of the DMN is observed only in an eyes-open condition (Mo et al., 2013). The role of alpha power in this context has been associated with its inhibitory role in blocking external visual input during introspective mental activity, which is not needed during the eyes-closed condition.

Oscillations in the gamma band, on the other hand, as defined in the above-mentioned model by Clayton et al. (2015), are associated with the excitation of task-relevant processes. This may be achieved by the rapid firing of interconnected neurons (falling into the 30–100 Hz frequency range that broadly encompasses gamma, Fitzgibbon et al., 2004), which would allow the sustained maintenance of information active in working memory or short-term memory (Jensen et al., 2007). This increment of gamma power, accompanied by a reduction in alpha power, in task-positive areas, has also been observed in intracranial EEG recordings (Ramot et al., 2012). In a complementary manner, intracranial EEG recordings have shown that gamma power is reduced in regions of the DMN during the performance of the CPT (Li et al., 2019). This shift away from task-irrelevant areas from gamma oscillations underscores the role of this neural signal, not only in areas relative to sensory processing but also in more complex cognitive functions. This has also been observed, not by absence, but by presence: as gamma power (orchestrated by and in feedback loops with theta power) in prefrontal regions has been associated with adequate conflict-detection, -resolution, and -adaptation (Oehrn et al., 2014). On the other hand, during tasks that are simpler or allow for an easier automatization throughout their performance, such as the PVT, a fluctuating role of gamma with time-on-task has been observed: a decrement with time-on-task, with a sharp pick-up to initial levels in the final block (Curley et al., 2023). This decrement of gamma power could reflect either automatization and thus, mindless execution, that requires less constant firing of task-relevant neurons, or, on the other hand, a depletion of resources that impedes this activation of task-relevant areas. The increment of gamma power toward the end of the task, which was accompanied by improved performance, may reflect the selective deployment of cognitive resources if a supervisory system detects that performance is not aligning with task goals (Curley et al., 2023). In fact, also Hemmerich et al. (2023) observed an increment of frontal gamma power with time-on-task in a vigilance task, which was further increased by the application of tDCS over the rPPC. Lastly, it is worth noting that whilst all the ranges defining narrow-band frequencies are somewhat arbitrary and vary between different studies (Cohen, 2021), this is especially accentuated in the gamma band, given its usually large span (30–100 Hz) and frequency filters used in EEG signal pre-processing, which may further hinder the integration between different studies, and especially those relating to the gamma band.

Furthermore, alpha and gamma oscillations also seem to interact, as oscillations alpha band gate those in the gamma band, as depicted in Figure 4C (Osipova et al., 2008). The pulsed inhibition of alpha power has been described to act as a “windshield wiper” mechanism, where this regular purging of task-irrelevant or distracting information, may play a crucial role in sustaining vigilance in accordance with task goals (Sadaghiani and Kleinschmidt, 2016). In fact, when inspecting neural oscillations on a trial-by-trial basis, Luna et al. (2023) observed that incorrect detections of an infrequent target were predicted by increased occipital alpha power before the target's onset. This increment of alpha power in task-relevant areas has been argued to be a potential contributor to the vigilance decrement (Luna et al., 2023); as it may reflect an imprecise deployment of this rhythmic inhibitory process that does not adequately serve task goals. Furthermore, interactions between oscillations in different frequency bands have also been explored in relation to vigilance performance, such as the task load index (ratio of parietal beta to the sum of parietal alpha and theta; Pope et al., 1995), the engagement index (ratio of frontal theta to parietal alpha; Kamzanova et al., 2014), the parietal alpha to frontal gamma ratio (Hemmerich et al., 2023), theta:beta ratio (Harty and Cohen Kadosh, 2019) among many others (Coelli et al., 2018; Hussain et al., 2021).

## 6 Conclusions and future perspectives

Vigilance has been studied for over 100 years, but yet we can still observe several gaps in our understanding of the phenomenon. Vigilance is hard to define and disentangle from other cognitive processes such as arousal, alertness, or sustained attention; and its decrement is explained by multiple—relatively contradictory—theories and associated with many different patterns of neural processes across myriad of (potentially) interacting regions. A unified theory that fully explains the vigilance decrement across different environments and tasks, as well as its varied manifestations and the best ways to counteract its effects, still remains elusive. Nonetheless, in this narrative review, we have attempted to disentangle a working definition of vigilance, differentiating it from other terms with which it is often used interchangeably. A proposed distinction is made, in terms of *intensity* (to separate it from sustained attention) and in terms of *direction* (to distinguish it from arousal and alertness), so that vigilance would be the ability to monitor the environment (specific direction) and detect rare but critical stimuli (low intensity).

Moreover, more critical accounts of the vigilance decrement have suggested that it may, in fact, be an iatrogenic phenomenon (Hancock, 2013). While such critiques importantly prompt reflection on the ecological validity of our methods, we maintain that vigilance—regardless of terminology—captures a relevant phenomenon that supports adaptive interaction with our environment. Still, this debate highlights the role of motivation and engagement, which differ meaningfully between lab and real-life contexts (e.g., experimental observation vs. safety-critical consequences). Recent work on tonic and phasic alertness further reinforces this view: although traditionally considered distinct processes, tonic alertness may provide a baseline for phasic fluctuations to become behaviourally meaningful (Poth, 2025). Crucially, both forms of alertness are increasingly seen as task-embedded and context-sensitive rather than purely bottom-up. For instance, phasic alerting effects depend on cue expectancy and are bounded by action sequences (Dietze et al., 2023), while indirectly modulating tonic alertness through postural change has shown to influence attentional performance (Barra et al., 2015). These findings suggest that vigilance, like alertness, is not a fixed latent capacity but a dynamic, emergent state, sensitive to be shaped by contextual, motivational, and task-driven factors. Future research into the potential sequential or bidirectional dependence of these different phenomena might aid in understanding each of their contributions better.

Moreover, we note that the present account does not fully disentangle different levels of description, such as abilities, functions, processes, and mechanisms, that may underlie vigilance and related constructs. In our conceptual overview, we primarily refer to vigilance as an ability; however, this cannot be meaningfully separated from the processes or computations (i.e., functions) required to sustain that ability over time. These, in turn, intersect with mechanisms—ranging from behavioral strategies to motivational dynamics—as described in various accounts of the vigilance decrement, as well as with the functional and structural neural correlates identified in the literature. While our terminology reflects conventions commonly used in the vigilance literature, future work may benefit from more explicitly mapping these levels of analysis onto observed phenomena.

In addition to the use of neuroimaging as described in the prior section, the use of direct assessments of resource consumption as well as self-reported mind-wandering, could help further elucidate the effects of what occurs “behind the scenes” of a vigilance task when modulating the different factors that affect vigilance (such as cognitive load, difficulty, engagement, among many others). Resource consumption could be recorded by assessing the brain's metabolic rate in response to the vigilance task through the use of near infrared spectroscopy (fNIRS, Borragán et al., 2018), and more causal hypotheses could be tested through the simultaneous use of NIBS and functional magnetic resonance imaging (Antal et al., 2011). On the other hand, a better grasp of mind-wandering during vigilance tasks might further help understand the phenomenon and its modulating factors. Finer-grained measures of mind-wandering could be obtained by simultaneous recording of direct but subjective self-reports (Weinstein, 2018) and other complementary objective but indirect measures of task engagement, such as eye movements (Krasich et al., 2018).

The complex intersection of motivational (Reteig et al., 2019) and emotional aspects (Shen et al., 2024), with cognitive load (Luna et al., 2022a; Pop et al., 2012) and task difficulty (Ballard, 1996; Helton et al., 2010; Thomson et al., 2015b), individual capacity (Caggiano and Parasuraman, 2004; Luna et al., 2022b), resources (Christie and Schrater, 2015), or individual differences in functional or structural brain connectivity (Esterman et al., 2013; Luna et al., 2021b; Rosenberg et al., 2016; Yamashita et al., 2021) potentially affecting task performance, should be explored in more detail in future research and could aid in developing more ecological assessments of the vigilance decrement (Chuang et al., 2018; Ma et al., 2020). This could ultimately support the transference of an in-lab validated understanding of vigilance and its decrement to real-life scenarios and clinical settings in a more straightforward manner.
